# Imaging and Treatment Features of Idiopathic Pancreatitis and Pancreas Divisum in a Young Man: A Case Report

**DOI:** 10.4021/gr529e

**Published:** 2013-07-14

**Authors:** Sari VenesmaaSari Venesmaa, Markku Heikkinen, Sakari Kainulainen, Hannu Manninen

**Affiliations:** a Department of Surgery, Kuopio University Hospital, Kuopio, Finland; b Department of Gastroenterology, Kuopio University Hospital, Kuopio, Finland; c Department of Clinical Radiology, Kuopio University Hospital, Kuopio, Finland; d Department of Clinical Radiology, Faculty of Health Sciences, Scholl of Medicine, Instute of Medicine, University of Eastern Finland, Finland

**Keywords:** Pancreas divisum, Idiopathic pancreatitis, Imaging, MRCP, ERCP

## Abstract

Some patients with pancreas divisum (PD) develop symptoms of recurrent pancreatitis. This is probably caused by insufficient drainage of the pancreatic duct. Magnetic resonance cholangiopancreatography (MRCP) is a non-invasive test reported to be highly accurate in diagnosing PD. Endoscopic minor papilla sphincterotomy is most effective in the treatment of patients with PD and pancreatic stones. We report a case of 17-year-old boy who has suffered from several abdominal pain attacks throughout his childhood without a specific diagnosis. Radiological findings after the first episode of pancreatitis were typical for PD and led to specific treatment and cure.

## Introduction

The pancreas develops from the ventral and dorsal buds, which later undergo fusion. Failure to fuse results in pancreas divisum (PD), which is defined by separate pancreatic ductal systems draining into the duodenum [1]. The issue of whether PD induces pancreatitis is controversial. However, Gonoi and co-workers concluded that PD should be considered as a predisposing factor for chronic and recurrent pancreatitis [2]. Endoscopic pancreatic stone removal via the minor duodenal papilla is a safe procedure to relieve pain [3]. We report a case of a young adult with PD and pancreatic stones in Santorini’s duct who presents with abdominal pain and acute pancreatitis.

## Case Report

A 17-year-old boy was admitted to our hospital with abdominal pain and dizziness that had started three days earlier. Previously, he has had five admissions to his own community hospital during the last three months with very similar symptoms. He also reported many pain episodes at home. In childhood he had suffered abdominal pain regularly and it was diagnosed as abdominal migraine. Gastroscopy was performed three years earlier with normal findings. Medical history was otherwise unremarkable. There was no alcohol abuse in his recent history. On admission, he had pain and tenderness in his upper abdomen but no signs of peritonitis or perforation. On physical examination, we observed T 36.9 °C, pulse 102/min (regular), blood pressure 148/104 mmHg. Laboratory tests: white blood cell count (WBC) 11.9 × 10^9^/L, hemoglobin (Hb) 162 g/L, C-reactive protein (CRP) 22 mg/L, plasma amylase (P-Amyl) 434 U/L and urine amylase (U-Amyl) 8,864 U/L. These amylase measurements were more than three times higher than normal values, confirming a diagnosis of pancreatitis with elevated CRP. Blood glucose, blood electrolytes, alanine transaminases, serum bilirubin, creatinine and urea measures were all within normal ranges. Respiratory and heart diseases were ruled out by chest radiography.

**Figure 1:**
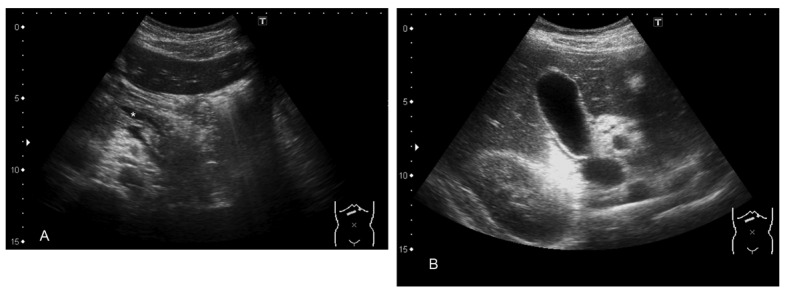
A) Pancreas and dilated duct (asterisk); B). Normal gallbladder.

On abdominal ultrasound, the pancreatic duct was abnormal. It was dilated from the corpus to the beginning of the tail by a length of 3 cm and to a width of 5 mm (Fig. 1A). There were no tumours in the pancreas. The liver, kidneys and spleen were normal. There were no stones in the gallbladder (Fig. 1B). MRI and MRCP were scheduled.

An abdominal MRI was performed with a 1.5 T MR scanner (Siemens Magnetom Avanto, Siemens AG, Germany) using phased-array body coils. A three-dimensional T2-weighted Space sequence (TR 3,344 ms; TE 690 ms; flip angle 140 degrees; 2 averages; resolution 1.0 × 1.0 × 1.0 mm^3^; interpolated in-plane resolution 0.5 × 0.5 mm^2^; 72 continuous coronal slices with FOV 380 mm; GRAPPA 3) with a navigator pulse was used for magnetic resonance cholangiopancreatography (MRCP). In addition to the original 1 mm slices, 40 mm MIP reformates and 3 mm MPR reformates were also made.

**Figure 2:**
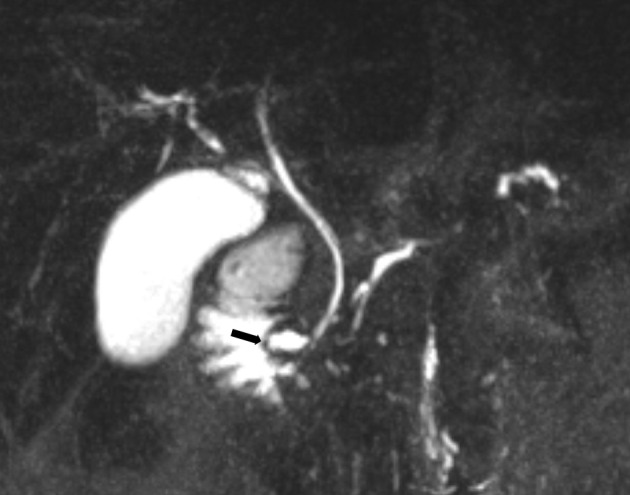
Pre-endoscopic treatment; 40 mm coronal MIP reformat of MRCP. The common bile duct runs to the major papilla. The pancreatic duct drains through the minor papilla (arrow).

**Figure 3:**
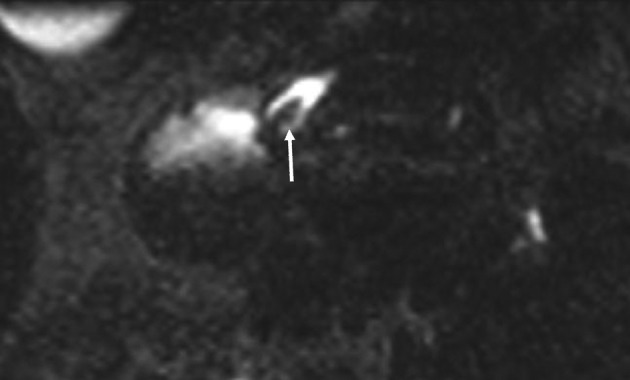
Pre endoscopic treatment; 3 mm axial MPR reformat through the minor papilla. The stone (arrow) is clearly visible in the ampulla of the accessory pancreatic duct.

**Figure 4:**
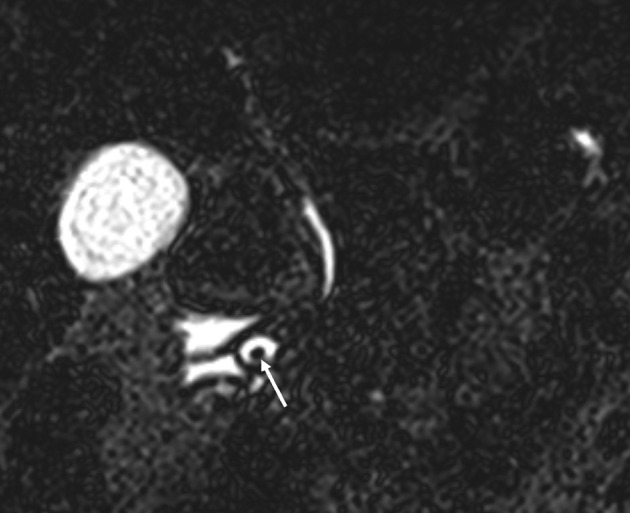
Original pre-treatment; 1 mm coronal MRCP-slice at the level of the minor papilla. The stone (arrow) in the ampulla on the accessory pancreatic duct is clearly visible.

The pancreatic duct in the pancreatic body and tail was irregular and slightly dilated. This duct was drained through an accessory pancreatic duct and minor papilla. Most of the accessory duct was very narrow but the last 1 cm of the duct just under the minor papilla formed a 6 mm wide ampulla containing a stone with a diameter of 8 × 3 mm. The common bile duct and the biliary tree were normal (Fig. 2-4)

**Figure 5:**
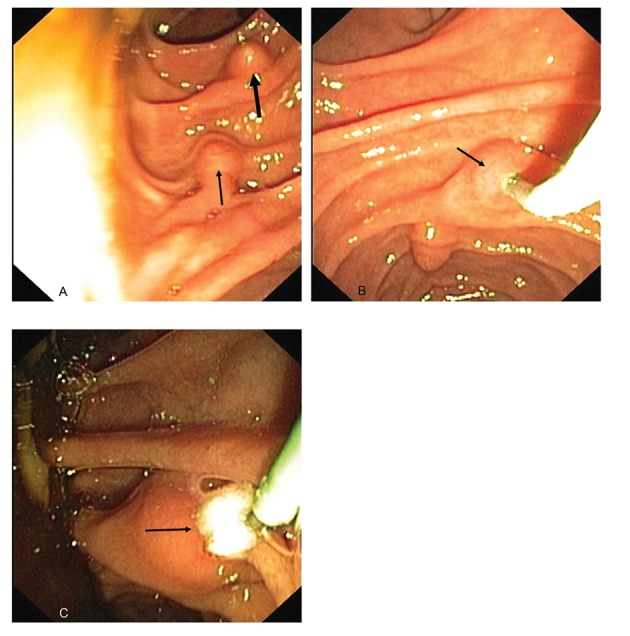
A). An endoscopic view from the papilla major (thick arrow) and more distal papilla minor (thin arrow); B). The minor papilla (arrow) was cannulated; C). The stone (arrow) was removed into the duodenum.

After MRCP, endoscopic retrograde cholangiopancreatography (ERCP) was performed. The procedure was started by two endoscopists after general anesthesia. The duodenoscopy (Olympus Evis Extra TJF-160VR) was transmitted to the duodenum where the papilla major and the more distal papilla minor were found (Fig. 5A). The minor papilla was cannulated by passage of a cannula (4.5 Fr) and ductography was done with a small amount of contrast injected slowly through the cannula (Fig. 5B). By radiography, the dorsal duct was seen but the scope was lying on the proximal part of it and so no stones were seen at first. The cannula was removed from the duct and at the same time a stone came out into the duodenum (Fig. 5C). The minor papilla was cannulated again and a guidewire was inserted. Standard papillotomy was made in approximately the 10 to 12 o’clock position. After papillotomy, a small quantity of sediment was removed from the duct and passed into the duodenum. The planned endoscopic treatment was performed successfully.

The patient recovered well and went home only seven hours after the endoscopic treatment. On the next day the laboratory tests were taken and there were no signs of post ERCP pancreatitis or any other complications. The MRCP control procedure was planned for one week later.

**Figure 6:**
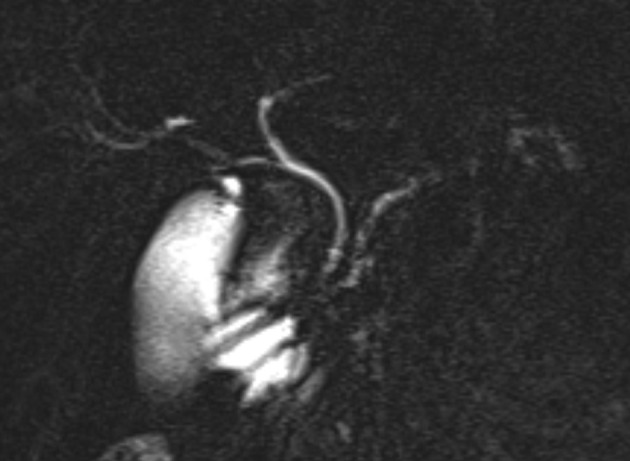
Post endoscopic treatment; 40 mm coronal MIP reformat of the MRCP. The ampulla of the accessory pancreatic duct is no longer visible.

After the intervention, the accessory pancreatic duct and the ampulla were no longer visible by MRCP (Fig. 6). Otherwise the findings of the pancreas and the biliary system were as described previously.

Six months after the endoscopic treatment the patient had not suffered from any kind of abdominal symptoms or pain attacks. There had been no need for any further hospital admissions after his treatment and stone removal from the pancreas divisum. This young adult was understandably relieved to be symptom free after such a long history of episodic abdominal pain.

## Discussion

Pancreas divisum is the most common congenital pancreatic anomaly, occurring in approximately 7 percent of people upon autopsy series [4]. More than 95 percent of patients with pancreas divisum are asymptomatic and it remains controversial whether the symptoms that occur in the remaining patients are etiologically related to pancreas divisum [4]. In some, the minor papilla orifice may be so small that excessively high intrapancreatic dorsal duct pressure occurs during active secretion, which may result in inadequate drainage, ductal distension, pain, and in some cases, pancreatitis [5]. Genetic studies have also suggested a multifactoral origin of pancreatitis in patients with pancreas divisum. As many as 10 to 20 percent of these patients carry at least one allele of the cystic fibrosis gene [6]. In our patient, the minor papilla orifice was almost completely occluded by ductal stones and the drainage was inadequate, resulting in dorsal duct distension that was seen in MRCP.

Acute abdominal pain is a common chief complaint in patients examined in the emergency department (ED) and can be related to a myriad of diagnoses. Obtaining a careful medical history and performing a thorough physical examination are the initial diagnostic steps for these patients. On the basis of the results of this clinical evaluation and laboratory investigations, the clinician will consider imaging investigations to help establish the correct diagnosis [7]. In one study, among 496 patients who presented to an ED with acute abdominal pain, the proportion of patients with a correct diagnosis after clinical evaluation increased from 70% to 83% after evaluation with US [8].

In our patient, US findings led to an alteration in treatment management. Instead of using computer tomography (CT) with ionizing radiation exposure, magnetic resonance (MR) imaging was performed. A standard CT of the pancreas may identify dilation of the dorsal duct and/or changes associated with chronic pancreatitis that are confined to the dorsal area of the pancreas. More commonly, the CT scan just shows nonspecific prominence of the pancreatic head and is no of diagnostic value [9]. Visualization of a fat plane between the dorsal and ventral portions can suggest pancreas divisum but does not generally separate symptomatic from coincidental states [10]. In a recent study, the diagnostic performance of multi-detector row computed tomography (MDCT) for the evaluation of PD was made using endoscopic retrograde pancreatography (ERP) as the reference standard. Pancreas divisum was correctly diagnosed in only 57% of cases [11].

The major advantage of MR imaging is the lack of ionizing radiation exposure. The high intrinsic contrast resolution rendered with MR imaging is another advantage, as intravenous contrast medium may not be required [7]. In acute pancreatitis, the role of MRCP is mainly limited to finding bile duct stones in patients with suspected biliary pancreatitis. MRCP is the method of choice for non-invasive assessment of the duct [1]. It is also reported to be highly accurate in diagnosing PD. However, based on the literature, MRCP without secretin is non-diagnostic for PD in a significant proportion of patients. Secretin-MRCP (S-MRCP) had a satisfactory specificity for detecting PD. However, the sensitivity of S-MRCP for the diagnosis of PD was modest at 73.3%. This is low compared to previous smaller studies, which reported a sensitivity of MRCP of up to 100% [12].

In our case the pancreatic duct was drained through an accessory pancreatic duct and minor papilla. The last 1 cm of the duct just under the minor papilla formed a 6 mm wide ampulla containing a stone bigger than that. The accessory duct was undoubtedly occluded. The hypothesis that obstruction of the minor papilla causes pancreatitis serves as the basis for performing drainage procedures of the duct of Santorini in patients with idiopathic pancreatitis and PD [13]. Recently, a study from India claims that relative obstruction at the minor papilla owing to PD plays a definite role in the pathogenesis of pancreatitis because endoscopic therapy in eight of 12 patients with PD had a 50% reduction per year in the frequency of attacks of pancreatitis [14]. Endoscopic sphincterotomy is receiving increased application for the opening of the minor papilla. The main indication for endoscopic sphincterotomy is in patients with recurrent episodes of acute pancreatitis [15]. Approximately 75 percent of patients with PD who have idiopathic acute recurrent pancreatitis improve after endoscopic therapy. Patients who have improvement show fewer pancreatitis attacks and hospitalizations [4]. In the largest series to date of patients with PD undergoing ERCP, the overall post-ERCP pancreatitis rate was just 6.8 percent. The possible complications and the small number of patients who need these procedures should limit this approach to select institutions with appropriate endoscopic expertise and high levels of endoscopic skills [3].

Whilst idiopathic pancreatitis is a rare cause of acute abdominal pain, many radiological tools are sometimes needed to find out the possible cause for this disease. With the development of imaging modalities and techniques, diagnostic accuracy for pancreas divisum has increased in patients with idiopathic pancreatitis. When taken together with a patient’s history and other examinations, the appropriate diagnosis and treatment can be found.

With this reported case, we present some clinical and radiological findings of pancreas divisum, which may bring attention to this condition and make future diagnosis and treatments more accurate and straightforward.
